# Assessing variability of the 24-hour pad weight test in men with post-prostatectomy incontinence

**DOI:** 10.1590/S1677-5538.IBJU.2014.0506

**Published:** 2016

**Authors:** Rena D. Malik, Joshua A. Cohn, Pauline A. Fedunok, Doreen E. Chung, Gregory T. Bales

**Affiliations:** 1 University of Chicago Medical Center, Chicago, IL, USA; 2 Mount Sinai Hospital, Chicago, IL, USA

**Keywords:** Urinary Incontinence, Suburethral Slings, Urinary Sphincter, Artificial, Stress

## Abstract

**Purpose:**

Decision-making regarding surgery for post-prostatectomy incontinence (PPI) is challenging. The 24-hour pad weight test is commonly used to objectively quantify PPI. However, pad weight may vary based upon activity level. We aimed to quantify variability in pad weights based upon patient-reported activity.

**Materials and Methods:**

25 patients who underwent radical prostatectomy were prospectively enrolled. All patients demonstrated clinical stress urinary incontinence without clinical urgency urinary incontinence. On three consecutive alternating days, patients submitted 24-hour pad weights along with a short survey documenting activity level and number of pads used.

**Results:**

Pad weights collected across the three days were well correlated to the individual (ICC 0.85 (95% CI 0.74–0.93), p<0.001). The mean difference between the minimum pad weight leakage and maximum leakage per patient was 133.4g (95% CI 80.4–186.5). The mean increase in 24-hour leakage for a one-point increase in self-reported activity level was 118.0g (95% CI 74.3–161.7, p<0.001). Pad weights also varied significantly when self-reported activity levels did not differ (mean difference 51.2g (95% CI 30.3–72.1), p<0.001).

**Conclusions:**

24-hour pad weight leakage may vary significantly on different days of collection. This variation is more pronounced with changes in activity level. Taking into account patient activity level may enhance the predictive value of pad weight testing.

## INTRODUCTION

Post-prostatectomy stress urinary incontinence (PPI) is reported to occur in up to one-third of patients (1–3). Depending on the degree of leakage and associated bother, many patients will seek definitive surgical management. Options for surgical management generally include male sling (MS) or artificial urinary sphincter (AUS) (4). Decision-making regarding surgical approach can vary significantly based upon the degree of leakage (5).

Practitioners commonly use pad usage per day to estimate the severity of urinary incontinence. While it is easy to determine by patient history, it is limited by variability in patient recall, description of type of pad use and correlation with actual urine loss (6, 7).

To objectively quantify urinary loss, pad tests ranging in duration from 20-min to 24-hours have been utilized (8, 9). 24-hour pad weights have been shown to be superior and are considered the gold standard for objective measurement of urinary incontinence (10, 11).

Previous authors have categorized incontinence into three categories based on the gram weight of urinary loss–mild PPI, or <100gm/24 hours, moderate PPI, or 100–400gm/24 hours, and high-grade, or >400gm/24 hours to help classify degree of incontinence (5). Furthermore, the severity of urinary incontinence, as determined by the 24-hour pad weight, has been shown to predict sling success or failure in previous studies (12–15). Specifically, a significantly decreased rate of success with MS in men with PPI >200gm/24 hours has been noted (13, 15, 16).

Typically, the 24-hour pad weight test is completed with no standardized activity instructions as recommended by the ICS (17). However, previous studies in women with stress urinary incontinence have shown variability in urinary loss associated with changes in activity level (18, 19). We aimed to address if similarly, activity level changes in men with PPI resulted in clinically significant changes in urinary loss using the 24-hour pad weight test. Our primary objective was to quantify changes in 24-hour pad weights over multiple days associated with alterations in activity level. Secondarily, we aimed to evaluate the correlation between self-reported number of pads, actual number of pads and 24-hour pad weights.

## MATERIALS AND METHODS

### Study subjects

Following institutional review board approval, we prospectively enrolled thirty-four patients presenting with PPI requiring the use of pads. All patients demonstrated comprehension of the study requirements and instructions and signed informed consent prior to enrollment. Patients spoke English and were able to confirm they had transportation available to go to the post office to mail pads and study items to the research staff. Patients were excluded from participation if they had previous surgery for incontinence, if they had any clinical urgency synmptoms, or if the answer to question 4 on the IPSS questionnaire “how often have you found it difficult to postpone urination?” was greater than 3.

### Data collection

At recruitment, patient's basic demographic information including age, date of radical prostatectomy, and self-reported number of pads used per day was collected. All patients were asked to complete three 24-hour pad weight tests on days 1, 3, and 5 following enrollment. Subjects were allowed to use as many pads as they deemed necessary and were provided sealable bags labeled day 1, 3, and 5 in which to store their soiled pads. Additionally, patients were asked to provide a dry pad. Subjects were asked to store their pads in the provided sealed bag in the refrigerator to minimize evaporation. Patients then mailed all pads back to the research staff via pre-stamped, pre-addressed United States Postal Service (USPS) Priority Mail® boxes. Once returned, number of pads used per day was counted. Each soiled pad was weighed using a calibrated scale. The weight of the dry pad was subtracted from each pad, and the final pad weights were summed to obtain the final 24-hour urinary leakage for each day.

On each day of 24-hour pad weight collection, patients completed a short survey documenting the number of pads used over 24-hours and activity level. In an effort to limit bias, patients were asked to complete this on the end of each individual day rather than at the completion of the study. Previously, when examining activity level in women with stress urinary incontinence, Painter et al. utilized a patient-completed activity diary to create three categories of activity (18). Adapted from their efforts we created a scale in which patients were asked to document activity level as: (0) sedentary, described as spending most of the day sitting, (1) mildly active, inclusive of no more than light housework, (2) moderately active, inclusive of light exercise or outdoor work, and (3) vigorously active, inclusive of strenuous aerobic exercise.

### Statistical analysis

An Intraclass correlation coefficient (ICC) was used to assess the correlation of three 24-hour pad weights to each individual patient relative to the other patient samples. The ICC is an index of reliability, which measures the agreement between variables (20, 21). When this correlation coefficient is close to 1, the variables tend to match and the reliability is high. Similar to other studies evaluating test-retest reliability, a cutoff of 0.7 was used (22). A sample size of 20 subjects with three observations per subject is 80% powered to detect an ICC of 0.7 or greater at an alpha of <0.05. The null hypothesis, i.e. that an individual's 24-hour pad weights were no more similar to his own than to those of the other subjects, would be accepted when the ICC is 0.4 or less using an F-test with a significance level of 0.05.

Paired t-tests were used to compare 24-hour pad weights and self-reported and actual pads used per day in each subject based on difference in self-reported activity level of no difference, one-point difference, or two-point difference. For example, a patient who identified himself as sedentary on day 1 (activity score=0) and moderately active on day 3 (activity score=2) would be considered to have a two-point increase in activity level.

Linear regression analysis was used to assess the relationship between self-reported pads per day at enrollment or actual collected pads per day with total 24-hour pad weight. All statistical analysis was performed using Stata v.12.0 (College Station, TX), with statistical significance considered for two-sided p-values of <0.05.

## RESULTS

Of the 34 patients consented for enrollment, 25 completed the study. Of the 9 patients who did not complete the study and were excluded from analysis, 6 patients did not return their pads and 3 patients returned pads, but had missing activity level data. Baseline patient demographics are summarized in Table-1. Mean age was 64.5±8.1 years and median time from prostatectomy was 33.9±42.3 months. Mean self-reported pad usage per day at enrollment was 3.0 pads (IQR 1.5–3.5 pads). On average, pads were received and weighed by research staff 14.0±7 days after enrollment.

**Table 1:** Patient Demographics.

Mean Age, years±SD	64.6±8.1
Mean BMI, kg/m^2^±SD	28±3.5
Months since surgery±SD	33.9±42.3
Mean days between recruitment and pads returned ± SD	14±7
Mean self-reported pads/day (IQR)	3.1 (1.5–3.5)

**Figure 1:**
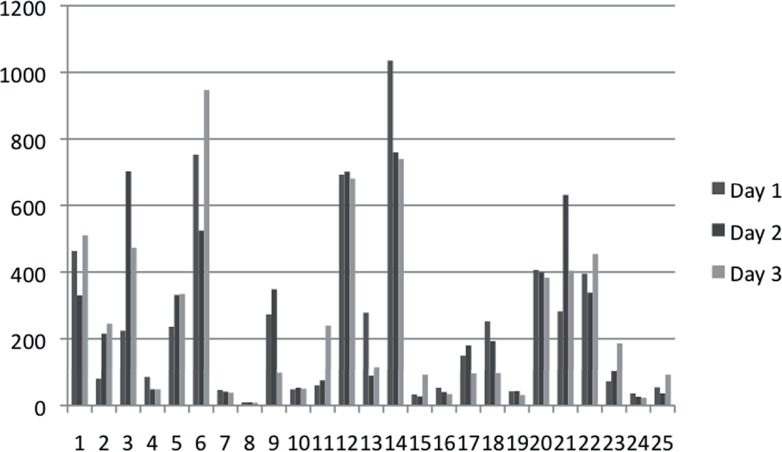
Three day 24-hour pad weights.

Mean 24-hour pad weight across subjects was 249.6±241.9g. The mean variability within each patient's 3 samples was 69.5g. The ICC for the cohort was 0.85 (95% CI 0.73–0.93, p<0.001) suggesting that despite any variability between collections, pad weights over three days were in general well correlated to each patient. Figure-1 is a graphic representation of twenty-four hour pad weights over three days for each subject.

### Activity level

Out of the 75 24-hour pad weight samples, activity level did not differ for 36 pairs, differed by 1 for 28 pairs, and differed by 2 for 11 pairs. With no change in activity level, pad weights varied by an average of 51.2g (95% CI 30.3–72.1, p<0.001). One-point and two-point increases in activity level were associated with mean increases in 24-hour pad weights of 118.0g (95% CI 74.3–161.7; p<0.001) and 138.8g (95% CI 35.3–242.3, p=0.01), respectively (Table-2).

**Table 2:** Variation in Activity Level & Mean Change in pad weights.

Change in Activity Level	Mean difference (g)	95% CI	*P-*value
No change	51.2	30.3, 72.1	<0.001
Change by 1 point	118.0	74.3, 161.7	<0.001
Change by 2 points	138.8	35.3, 242.3	0.01

### Pads per day

**Figure 2:**
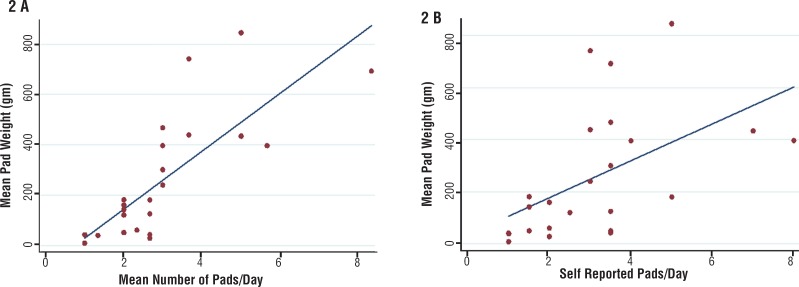
A. Change in mean 24-hour pad weight by mean number of pads used per day.B. Change in mean 24-hour pad weight based on self-reported pads used per day.

On linear regression analysis, each one-unit increase in self-reported pads per day at enrollment was associated with a mean pad weight increase of 72.1g (95% CI 23.0–121.2, p=0.006). For each 1-unit increase in collected pads, mean pad weight increased by 114.1g (95% CI 74.8–153.4, p<0.001). Patients self-reported using more pads than actually collected by an average of 0.85 pads per day (95% CI 0.41–1.30, p<0.001) (Table-3). Figure-2 is a graphical representation of change in mean pad weight by mean number of pads used and self-reported pads used per day.

**Table 3:** Association of Mean Pad Weight & Mean Number of Pads.

Increase in Pad Weight (g)		95% CI	*P*-value
Per Mean Pads/Day	114.1	74.8, 153.4	<0.001
Per Self Reported Pads/Day	72.1	23.0, 121.2	0.006

Based on change in activity level, pad usage also varied. With no change in activity level, pad number varied by 0.47 (95% CI 0.24–0.71; p<0.001). One-point and two-point increases in activity level were associated with an increase in pad number by 0.71 (95% CI 0.40–1.0; p<0.001) and 0.82 pads per day (95% CI 0.41–1.2; p=0.001), respectively (Table-4).

**Table 4:** Variation in Activity Level & Mean Change in Number of Pads.

Change in Activity Level	Mean change in pads	95% CI	P-value
No change	0.47	0.24, 0.71	<0.001
Change by 1 point	0.71	0.40, 1.0	<0.001
Change by 2 points	0.82	0.41, 1.2	0.001

## DISCUSSION

To our knowledge, this is the first study aiming to objectively quantify the variability of urinary leakage in men with PPI. We find that multiple 24-hour pad weights in men with PPI correlate well with a particular individual within a sample patient population based on the ICC. However, variation in activity level can lead to significant differences in 24-hour pad weights between days. In our cohort, an increase in activity level by one or two points resulted in a statistically significant increase in 24-hour pad weight by over 118g and 138g. Similarly, in women, increased activity levels have been associated with increased severity of urinary incontinence. In a prospective study of 25 women with isolated clinical stress urinary incontinence, Painter et al. demonstrated significant variability in pad weight leakage based upon self-reported activity level and subsequently recommended the implementation of standardized activity instruction for patients completing the 24 hour pad weight test (18). In another study in which women with isolated stress urinary incontinence submitted 7-consecutive 24-hour pad weights, leakage between days varied by an average of 22.6g (19).

The volume of pre-operative urinary leakage has been suggested to be the main predictor of a successful outcome with surgical interventions for male post-prostatectomy incontinence (12, 16). The 24-hour pad weight test is the gold standard for objective measurement of urinary incontinence and continues to be an important aspect of pre-operative evaluation to determine optimal treatment recommendations. However, as seen in our cohort, changes greater than 100gm can be seen in patients with variation in daily activity level.

In an effort to improve clinical utility and consistency, the ICS Urodynamics Committee has recommended the use of the 24-hour pad weight with instructions for patients to perform their normal daily routine rather than standardized physical activity (17). However, it remains important to note that patients may have days on which they are more active than others. It may be informative to ask patients to behave at their maximum activity level, or how active they would be if they were continent. This may permit a more accurate evaluation of the highest volume of incontinence the patient can experience. While the 24-hour pad weight test is considered the gold standard to quantify urinary leakage, recent surveys have found that only 4.5–8.0% of physicians routinely perform pad weight testing, and up to one fourth of physicians never administer pad weight testing to their patients (23). The labor involved in pad weight testing is difficult for patients, as well. In response, considerable effort has been devoted to identify effective but less burdensome means of quantifying urinary incontinence (24).

Clinicians often use pad usage per day as a measure to describe incontinence. In our cohort, we have found that collected pads per day as well as self-reported pads per day are associated with significant increases in mean pad weight albeit with wide confidence intervals. This can be used in clinical practice, however, it remains important to query how often patients change pads and what degree of wetness they subjectively note when changing pads to assess whether they might fall towards the upper or lower limit of the 95% confidence interval identified in our study (23–121gm). In an earlier study, number of pads collected during a 24 hour pad weight test was also shown to be a reliable measure of objective incontinence (22). Conversely, Dylewski et al. compared the number of pads per day with the 24-hour pad weight test and found that self-reported pads per day was not a reliable measure of incontinence (6). Likely self-reported pads are one aspect of the assessment that is helpful but does not definitively or adequately quantify leakage.

In combining pads per day with a 4-item questionnaire, Nitti et al. found that patient's description of the number, size, and degree of wetness of pads over a 24 hour period and its impact on their quality of life correlated well with their 24-hour pad weight (24). This may be useful in patients who are non-compliant with 24-hour pad testing or in cases where it is not feasible. Further studies are required to validate these findings and correlate them with treatment outcomes.

Our study is limited due to several factors that deserve attention. Nine of our initially enrolled thirty-three patients (27%) were excluded from analysis due to noncompliance with study instructions or inability to return pad weights. Within the study parameters, we were able to recruit patients who demonstrated understanding of the study instructions, spoke English, and had transportation available to go to the post office to mail pads and study items to the research staff, which may limit the generalizability of the data to the general population. We are limited by a possible evaporative effect on the pad weight. However, we asked patients to place their used pads in a provided sealed bag in the refrigerator prior to mailing. Additionally, Versi et al. found that sealed pads had statistically insignificant losses of pad weight due to evaporation up to eight weeks after pads were sealed (25). With regard to activity level, we relied on self-report with a subjective survey, not allowing for standardization between patients. However, comparisons were made of data from the same patient rather than between patients, hopefully limiting the inherent limitations with the use of a survey questionnaire. We are further limited by our results which also revealed a statistically significant change in pad weight in those patients who reported no change in activity level. In addition to activity, fluid intake can also significantly impact degree of urinary leakage.

## CONCLUSIONS

Variability in 24-hour pad weights is significantly increased with changes in activity level. Attention to physical activity level may be helpful in better characterizing urinary incontinence. Further studies with a larger number of subjects are required to validate these findings.
